# Becoming Self-Aware of Feelings and Performance: The Influence of Creative Potential, Self-Evaluations, and Metacognitive Feelings on Creative Mindsets

**DOI:** 10.3390/jintelligence11070138

**Published:** 2023-07-12

**Authors:** Rogelio Puente-Díaz, Judith Cavazos-Arroyo, Lizbeth Puerta-Sierra

**Affiliations:** 1 School of Business and Economics, Universidad Anahuac México, Av. Universidad Anáhuac 46, Huixquilucan C.P. 52786, Mexico; 2 Centro Interdisciplinario de Posgrados, Universidad Popular Autonoma del Estado de Puebla, Puebla C.P. 72410, Mexico; judith.cavazos@upaep.mx

**Keywords:** creative potential, metacognitive feelings, mindsets

## Abstract

Based on a recently developed model of creative cognition, we tested in two studies, the relationships between creative potential, self-evaluations, metacognitive feelings, and growth and fixed mindsets in creative action. In both studies, participants (N = 491, mean = 21.57, SD = 2.78 and N = 280, 94% between the ages of 18 and 25 years, respectively, for studies 1 and 2) first completed a divergent thinking task, followed by an assessment of metacognitive feelings, self-evaluations of the creativity of the ideas generated (only in study 2), and creative mindsets while knowing that a second divergent thinking task was coming. Results showed that creative mindsets were sensitive to variations in creative potential, self-evaluations, and metacognitive feelings when examined in creative action. Specifically, studies 1 and 2 showed positive relationships between metacognitive feelings and growth mindsets. Results from study 2 showed positive relationships between self-evaluations of the idea generated and growth mindsets. For fixed mindsets, the creative potential of task 1 had a negative relationship in study 1 and a negative relationship between fixed mindsets and the creative potential of task 2 in study 2. The implications for creative metacognition were explored.

## 1. Introduction

Creative metacognition has received more attention in recent years (Puente-Díaz 2023). Yet, progress has been slow partly due to the lack of a comprehensive model of creative metacognition. This situation is likely to change with a recently developed model of creative metacognition (Lebuda and Benedek 2023). We would like to take this opportunity to test some of the components of this model along with how creative mindsets interact with some indicators of metacognition. Thus far, the attention paid to how different indicators of metacognition interact has been limited (Lebuda and Benedek 2023). Similarly, the attention given to how different metacognitive indicators influence creative mindsets has been even more limited. We seek to address this limitation through focusing on how creative potential, self-evaluations of ideas generated, and metacognitive feelings inform creative mindsets, consistent with a conceptualization of a mindset as a creative awareness (Karwowski et al. 2019a). We test these possibilities using two studies. Hence, the purpose of the present investigation is twofold. First, we test in one study how creative potential and creative metacognitive feelings inform creative mindsets and the relationships between creative mindsets and subsequent creative potential. Second, we test in study 2 how, in addition to creative potential, self-evaluations of the ideas generated inform creative mindsets. To reach our research goals, we briefly discuss how metacognition has been conceptualized in creativity, followed by a discussion of creative mindsets.

### 1.1. Guiding Principles

To guide our research efforts, we take a situated cognition perspective to explain how metacognitive indicators inform creative mindsets. A core principle of situated models of cognition and problem-solving is that thinking is for doing (Newen et al. 2020). What this entails is that beliefs and judgments are malleable and adjust to context. Taking a situated cognition perspective and combining it with recent developments in creative metacognition (Lebuda and Benedek, preprint), we postulate three guiding principles: (1) Metacognitive indicators such as feelings and self-evaluations inform creative mindsets. Creative potential, as rated by independent judges, also represents a source of information for creative mindsets. The main reason for this proposition is because (2) creative mindsets represent creative awareness (Karwowski et al. 2019c) and knowledge structures (Leith et al. 2014; Plaks and Stecher 2007) capable of adjusting after receiving relevant feedback. From the work on metacognition in memory research (Koriat and Goldsmith 1996), we know that (3) beliefs and judgments could be formed based on theory-driven or data-driven sources or antecedents. We use these principles to guide our exploratory research efforts to examine how self-evaluations of the ideas generated, metacognitive feelings, and creative potential inform creative mindsets when examined in creative action.

To test our exploratory ideas, we conceptualize creative action as the assessment of creative mindsets after performing an idea generation task and being aware of a second divergent thinking task ahead. Being aware of a second task makes the assessment of creative mindsets situated and with greater relevance. We contrast creative action with a creative inaction approach in which the assessment of mindsets is not contextualized, preventing participants from using objective creative potential, self-evaluations, or metacognitive feelings as sources of information. Thus far, more attention has been given to the assessment of creative mindsets under scenarios of creative inaction.

### 1.2. Creative Metacognition

The creative process involves cognitive and metacognitive mechanisms interacting and informing each other (Puente-Díaz and Cavazos-Arroyo 2020; Sidi et al. 2020). A recent model developed by Lebuda and Benedek (2023) suggests that metacognitive knowledge, monitoring, and control interact during the preparation, generation, evaluation, and selection of creative ideas. As individuals generate ideas, they need to answer a basic, difficult question regarding the potential originality and effectiveness of their ideas. The criteria of originality and effectiveness are more ambiguous, making the process of idea generation, evaluation, and selection more difficult as compared to problems with correct solutions (Ackerman 2019; Puente-Díaz and Cavazos-Arroyo 2020; Sidi et al. 2020). We suggest that self-evaluations and metacognitive feelings are two relevant indicators of how well the idea-generation process is going. These two indicators have been examined in previous studies of metacognition (Puente-Díaz and Cavazos-Arroyo 2022). Self-evaluations inform the process of idea generation and selection. It is metacognitive because individuals evaluate their own cognitive outputs (Puente-Díaz 2023). Even though creative potential is not metacognitive, it represents another source of information. Creative potential represents another important source to inform the creative process, and it has often been conceptualized as performance on a divergent thinking task (see Runco and Acar 2019). For example, the serial order effect found in previous investigations (Beaty and Silvia 2012; Sidi et al. 2020) suggests that individuals monitor and control the creativity of the ideas they generate even if they are not asked to explicitly do so. Consequently, we suggest that self-evaluations, metacognitive feelings, and creative potential inform creative mindsets in a way that has not been explored before. Now we turn to a discussion of creative mindsets.

### 1.3. Creative Mindsets as Knowledge Structures

Creative mindsets represent beliefs or judgments about the malleability—growth mindsets—or stability—fixed mindsets—of creative performance and skills (Karwowski 2014). The work on creative mindsets comes from the development of lay beliefs or theories of intelligence (Dweck 1999). As suggested by a recent literature review and conceptual developments, creative mindsets might not be a strong predictor of creative performance but a variable that mediates the relationship between creative potential and creative outcomes (Hass et al. 2019; Karwowski et al. 2019a; Royston and Reiter-Palmon 2019). We agree with this conceptualization but would like to add that creative mindsets are likely to respond to changes in creative performance and to how individuals feel about their performance, consistent with a recent conceptualization of creative mindsets as creative awareness (Karwowski et al. 2019a) and with a recent model of creative metacognition (Lebuda and Benedek 2023). The idea of conceptualizing creative mindsets as sensitive knowledge structures might help increase specificity in the examination of mindsets, which has been a limitation in previous research (Royston and Reiter-Palmon 2019). In other words, individuals do not only think about the malleability or stability of creative performance and skills spontaneously (theory-driven judgments) but also in response to their objective and perceived performance and their feelings about it (data-driven judgments). This proposition comes from the idea that mindsets represent knowledge structures that change their levels of activation in different contexts (Leith et al. 2014; Plaks and Stecher 2007). That is, individuals adjust, to some extent, their beliefs about the stability or malleability of creative performance and they are more likely to do so after performing a creative task. This represents a situated cognition approximation to examining creative mindsets, which, to our knowledge, has received limited attention in creativity research. Previous research has usually examined creative mindsets in isolation from cognitive and metacognitive indicators of creative potential (Karwowski et al. 2019a). This is a limitation we seek to address through assessing creative mindsets in creative action.

Specifically, performing a creative task would make individuals aware of their performance and, consistent with situated models of cognition and problem-solving, more likely to adjust their beliefs accordingly even without receiving explicit feedback from an external source. This prediction is in line with the idea that cognition is affordance-based, where affordances are relational in nature (Newen et al. 2020). In the absence of external feedback, individuals could use metacognitive feelings and implicit and explicit evaluations of their creative performance to adjust their beliefs about the malleability or stability of creative abilities and performance. Empirical evidence shows that mindsets are malleable (Karwowski et al. 2019b), trainable (Mrazek et al. 2018), and sensitive to goals (Leith et al. 2014). Consequently, they might adjust to variations in creative performance and their corresponding feelings. To our knowledge, previous studies have not tested the propositions just explained.

Given that both conceptualizations, i.e., creative mindsets as knowledge structures and creative awareness, are relatively new, there is not enough empirical evidence to posit specific hypotheses. Hence, we explore the possibility that creative performance or potential, as assessed by independent judges and via self-evaluations, and metacognitive feelings would be related to growth and fixed mindsets when creative mindsets are examined in creative action, that is, when individuals perform an idea generation task and make inferences about their performance.

In sum, the purpose of the present investigation is twofold. First, we test in one study how creative potential and creative metacognitive feelings inform creative mindsets and the relationships between creative mindsets and subsequent creative potential. Second, we test in addition to creative potential how self-evaluations of the ideas generated inform creative mindsets.

### 1.4. Overview of Studies

Given that we wanted to examine creative mindsets in creative action, we needed to do the following: First, we needed to assess creative mindsets after performing a divergent thinking task while knowing that a second divergent thinking task was ahead. This was our creative action design because the assessment of creative mindsets came after performing a creativity task and before participants had the chance of performing a second creativity task. We conducted two studies of creative action that complemented each other. The first study focused on creative potential and metacognitive feeling while the second study also included self-evaluations of the creativity of the ideas generated.

## 2. Study 1

### 2.1. Material and Method

#### 2.1.1. Participants and Procedure

Participants were 491 college students (62% females, ages ranging from 18 to 31, mean = 21.57, SD = 2.78) with an educational background in business, where students are often required to generate ideas to solve business problems. Participants were informed that they were about to participate in a study dealing with the generation of ideas to solve business problems. In addition, participants were told that after completing the first idea-generation task and answering some questions, they were going to participate in a second idea-generation task. These instructions, presented at the beginning of the study, were important because we wanted participants to be aware of a second idea-generation task. Participants completed the full battery of questionnaires in 15 min. The internal human subjects committee of our universities approved the study.

#### 2.1.2. Measures

After reading the instructions informing about participation in two divergent thinking tasks and answering a set of questionnaires, participants were introduced to the following problem and asked to generate three ideas:

The company Apple is one of the most successful in the world. However, in the last quarter of 2018, Apple reported lower sales than expected, especially for its new iPhones: iPhone XS and XS Max. In addition, the forecasts for the first quarter of 2019 are not optimistic. We want you to think that Apple has just hired you as a consultant and your first task is to generate ideas to revert the decline in sales. What do you think it can be done? Your ideas could represent many things including product improvements, design, new colors, Apps, positioning, price, distribution, and advertising, among others. Generate three novel and useful ideas and write them in the space below please.

Previous studies have used similar business problems to assess creative potential (Puente-Díaz et al. 2021). After generating ideas, participants answered questions about ease of idea generation, using a 6-item validated questionnaire that uses a scale ranging from 0 (definitely no) to 100 (definitively yes) (Puente-Díaz et al. 2021). A recent review validated the importance of metacognitive feelings as assessed with this scale (Lebuda and Benedek 2023), yet more empirical work is needed. The scores showed acceptable levels of reliability (h = .91). In addition, participants rated the creativity of their best idea on a scale from 0 (not creative at all) to 10 (very creative) and ranked their ideas from the most creative (first place) to the least creative (third place). Then, participants completed the 10-item creative mindset scale (Karwowski 2014). The scores for growth and fixed mindsets showed acceptable levels of reliability (h = .70 and h = .70, respectively). We made minor modifications to the wording to make items more specific to our context. For example: Everyone can generate great business ideas at one point if he or she is given appropriate conditions, and anyone can develop their abilities to generate business ideas up to a certain point, among other changes.

Participants were then introduced to a second problem and were asked to generate three ideas. The problem was:

Mexico has a serious problem of frequent floods and the main causes of these floods are litter that covers the drains. The amount and variety of litter that is thrown away are significant, from food wrappers and containers to cigarette butts. We would like you to think that you have been just hired to act as a consultant and your first task is to generate ideas to create a campaign to reduce litter on the streets. What do you think it can be done? Generate three novel and useful ideas and write them in the space below please.

After generating ideas, participants rated the creativity of their best ideas on a scale from 0 (not creative at all) to 10 (very creative) and completed some demographic questions. Hence, the key feature in our design was the acknowledgment from the beginning that the complete package included an idea-generation task, followed by some questions, and then a second idea-generation task. Two independent judges evaluated all the ideas generated by participants in task 1 and task 2 using the same scale from 0 (not creative at all) to 10 (very creative). Scores showed acceptable levels of interrater reliability (rwg = .80 and rwg = .83, respectively, for tasks 1 and 2).

### 2.2. Results

#### 2.2.1. Overview of Analytical Strategy

We used structural equation modeling with Mplus 7.11 treating all variables as latent. In addition, some variables were treated as having a normal distribution and others as having a non-normal distribution. In both studies, we report a combination of absolute and incremental fit index: χ^2^, Root Mean Square Error of Approximation (RMSEA), Comparative Fit Index (CFI), and Tucker–Lewis Index (TLI). We used the cutoff scores of RMSEA = < .08 and CFI and TLI > .90 as the minimum acceptable levels of model fit (West et al. 2012).

#### 2.2.2. Structural Equation Model

The measurement model included five latent variables: creative potential assessed by judges of task 1 (the mean of the score of two judges for three ideas), metacognitive feelings (six items), growth and fixed mindsets (five items each) and creative potential assessed by judges of task 2 (the mean of the score of two judges for three ideas). Results for the measurement model showed an acceptable model fit with χ^2^ = 291.188, *p* < .001 (df = 220), RMSEA = .03, CFI = .95, and TLI = .94. Examination of the individual parameters revealed that all factor loadings were significant and in the expected direction (ranging from .10 to .82). The bivariate correlations between the latent variables were moderate to strong, ranging from −.02 to .70. Given the results of our measurement model, we can conclude that the scores of the latent variables were reliable (reported in the Measures section) and valid to test our hypotheses. Consequently, we proceeded to test our structural model.

**Figure 1:**
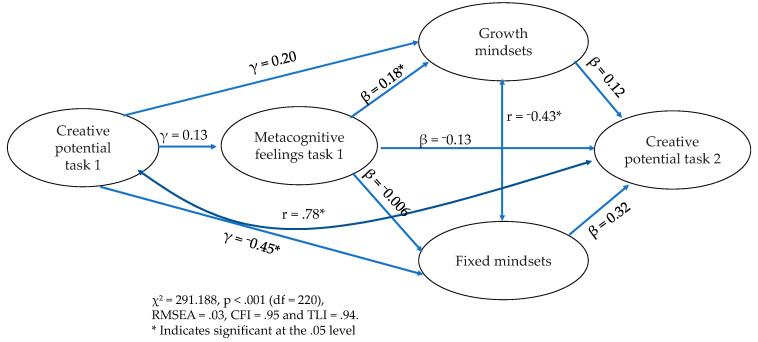
Standardized parameter estimated for model 1.

Results for the structural model showed an acceptable model fit with χ^2^ = 291.188, *p* < .001 (df = 220), RMSEA = .03, CFI = .95, and TLI = .94. Examination of the individual parameters showed that the relationship between creative potential of task 1 and metacognitive feelings was not significant: γ = .13, *p* = .28. Regarding growth mindsets, whereas metacognitive feelings had a significant positive relationship (β = .18, *p* =. 01) the creative potential of task 1 did not have a significant relationship (β = .20, *p* = .21). For fixed mindsets, the creative potential of task 1 had a significant, negative relationship, with β = −.45 and *p* <. 001. Conversely, the relationship between metacognitive feelings and fixed mindsets was not significant, with β = −.006 at *p* = .94. We did not find any significant predictors of creative potential in task 2. Neither metacognitive feelings (β = −.13, *p* = .46) nor growth mindsets (β = .12, *p* = .62) or fixed mindsets (β = .32, *p* = .41) had significant relationships. The correlations (without indicating directionality) between both mindsets and between the creative potential of task 1 and creative potential of task 2 were r = −.43, *p* < .001 and r = .78, *p* < .001, respectively (see Figure 1).

### 2.3. Brief Discussion

Our results showed preliminary evidence for the idea that beliefs about the malleability or stability of creativity abilities were sensitive to fluctuations in creative potential and metacognitive feelings, consistent with models of situated problem-solving (Newen et al. 2020). Given the exploratory nature of our first study, it was important to conduct a conceptual replication. Consequently, in our next study, we replicated most of the characteristics of the design of study 1 but added self-evaluations as an additional source of information for creative mindsets.

## 3. Study 2

### 3.1. Material and Method

#### 3.1.1. Participants and Procedure

Participants were 280 college students (62% females, 28) with an educational background in business. Participants received the same instructions as in study 1. Participants completed the full battery of questionnaires in 15 min. The internal human subjects committee of our universities approved the study.

#### 3.1.2. Measures

As in study 1, after reading the instructions informing about participation in two divergent thinking tasks and answering a set of questionnaires, participants were introduced to the following problem and asked to generate three ideas:

Most of us are familiar with cell phones or smartphones. We are going to ask you to generate ideas to improve cell phones. The ideas have to be original (uncommon) and effective (solve a consumer problem). Generate three original and effective ideas and write them in the space below please.

As in study 1, participants completed the 6-item measure of ease of idea generation (Puente-Díaz et al. 2021). The scores showed acceptable levels of reliability (h = .95). In addition, participants were asked to self-evaluate their ideas on originality and effectiveness using a scale from 0 (not original/effective at all) to 10 (very original/effective) (h = .85) and completed the 10-item creative mindset scale (Karwowski 2014). The scores for growth and fixed mindsets showed acceptable levels of reliability (h = .78 and h = .79, respectively).

Participants were then introduced to a second problem and were asked to generate three ideas. The problem was:

Most of us are familiar with the company Apple. For this task, we are going to ask you to generate ideas to increase the number of subscriptions to its Apple TV+ service. The ideas have to be original (uncommon) and effective (solve a consumer problem). Generate three original and effective ideas and write them in the space below please.

After generating ideas, participants completed some demographic questions. Two independent judges evaluated all the ideas generated by participants in task 1 and task 2. Scores showed acceptable levels of interrater reliability (rwg = .77 and rwg = .80, respectively, for tasks 1 and 2; see Table 1 for a summary of sequence of procedure).

### 3.2. Results

The measurement model included six latent variables: creative potential assessed by judges of task 1 (the mean of the score of two judges for three ideas), metacognitive feelings (six items), self-evaluations of task 1 (two ratings of each of the three ideas generated for a total of six), growth and fixed mindsets (five items each), and creative potential assessed by judges of task 2 (the mean of the score of two judges for three ideas). Results for the measurement model showed an acceptable model fit with χ^2^ = 795.31, *p* < .001 (df = 309), RMSEA = .08, CFI = .95, and TLI = .95. Examination of the individual parameters revealed that all factor loadings were significant and in the expected direction (ranging from .33 to .92). The bivariate correlations between the latent variables were small to strong, ranging from .001 to .75. Given the results of our measurement model, we can conclude that the scores of the latent variables were reliable (reported in the Measures section) and valid to test our hypotheses. Consequently, we proceeded to test our structural model.

**Figure 2:**
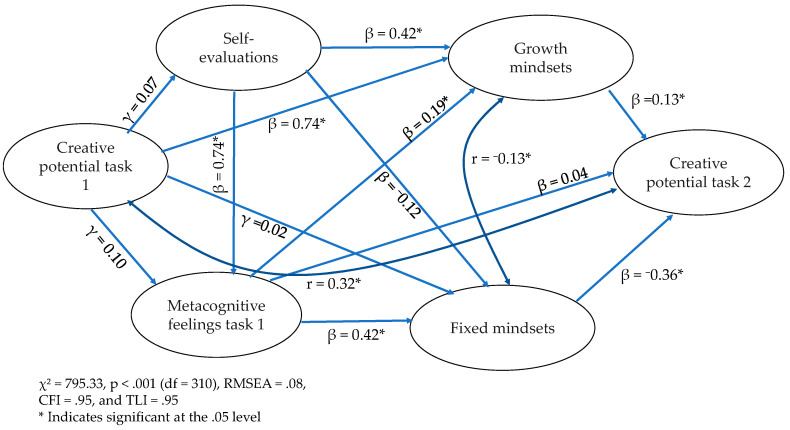
Standardized parameter estimated for model 2.

Results for the structural model showed an acceptable model fit with χ^2^ = 795.33, *p* < .001 (df = 310), RMSEA = .08, CFI = .95, and TLI = .95. Examination of the individual parameters showed that the relationship between the creative potential of task 1 and metacognitive feelings was not significant, with γ = .10 and *p* = .17. Self-evaluations had a positive relationship with metacognitive feelings (γ = .74, *p* < .001) and a non-significant relationship with creative potential (γ = .07, *p* = .23). Regarding growth mindsets, whereas metacognitive feelings and self-evaluations had significant positive relationships (β = .19, *p* = .03; β = .42, *p* < .001), the creative potential of task 1 did not have a significant relationship (β = −.05, *p* = .45). For fixed mindsets, neither the creative potential of task 1 nor self-evaluation had significant relationships (β = .02, *p* =.80; β = −.12, *p* =.30). Conversely, the relationship between metacognitive feelings and fixed mindsets was significant, with β = .42 and *p* < .001. We found one significant predictor of the creative potential of task 2, fixed mindsets, with β = −.36 and *p* < .001. Conversely, neither metacognitive feelings (β = .04, *p* = .76) nor growth mindsets (β = .13, *p* = .22) had significant relationships with the creative potential of task 2. The correlations (without indicating directionality) between both mindsets and between the creative potential of task 1 and creative potential of task 2 were r = −.13, *p* = .044 and r = .32, *p* = .003, respectively (see Figure 2 and Table 2 for a summary of results).

### 3.3. Brief Discussion

Whereas the negative relationship between the creative potential of task 1 and fixed mindsets was not replicated, we observed a significant relationship between fixed mindsets and the creative potential of task 2. Similarly, metacognitive feelings and self-evaluations of the ideas generated had a significant positive relationship with growth mindsets. Hence, our idea of assessing mindsets in creative action received mixed support. We also found a positive relationship between metacognitive feelings and fixed mindsets, which was unexpected.

## 4. General Discussion

The purpose of the present investigation was twofold. First, we tested in one study how creative potential and creative metacognitive feelings informed creative mindsets and the relationships between creative mindsets and subsequent creative potential. Second, we tested how, in addition to creative potential, self-evaluations of the ideas generated informed creative mindsets. Results showed that creative mindsets were sensitive to variations in creative potential, self-evaluations, and metacognitive feeling when examined in creative action, with relevant implications for advancing our understanding of creative metacognition and mindsets and for the development of creative skills in education.

### 4.1. Creative Metacognition: The Role of Creative Mindsets

We made three important claims in our introduction: (1) Creative mindsets represent a form of creative awareness, where individuals hold lay beliefs about the malleability or stability of creative potential (Karwowski et al. 2019a). Creative mindsets represent a form of knowledge structure that becomes activated when needed. (2) Creative mindsets could be informed by theory-driven antecedents or data-driven antecedents (Koriat 2006). Regarding data-driven antecedents, creative potential as rated by independent judges, metacognitive feelings, and self-evaluations could inform creative mindsets, but only when mindsets are assessed in creative action. In other words, they would only represent a source of information when individuals first engage in a divergent thinking task while knowing that a second task would come after assessing mindsets. (3) Two metacognitive indicators, self-evaluations and metacognitive feelings and creative potential, should inform creative mindsets, as suggested by a recently developed model of creative metacognition (Lebuda and Benedek, preprint). Overall, we found partial support for these three claims.

First, we tested the correlations between metacognitive feelings and growth mindsets in two studies. The correlations were significant when individuals completed the assessment of growth mindsets after performing a creative task and reporting their metacognitive feelings while knowing that a second creativity task was coming. This provides preliminary support for the idea that growth mindset judgments could be data-driven because metacognitive feelings were used as information. These findings were consistent with the idea that feelings are part of metacognition, which informs beliefs and judgments in the form of creative mindsets (Lebuda and Benedek, preprint).

Regarding fixed mindsets, results from study 2 showed a positive relationship with metacognitive feelings, which was unexpected, and non-significant relationships in study 1. Whereas the findings for growth mindsets were consistent with our conceptual developments, the findings for fixed mindsets were somewhat surprising given that we expected a negative relationship between fixed mindsets and metacognitive feelings.

Self-evaluations of the ideas generated represented another source of information for growth and fixed mindsets. Self-evaluations only had a positive relationship with growth mindsets, suggesting that they could be considered a relevant source of information. While the relationship between self-evaluations of the idea generated and fixed mindset was in the expected direction, negative, it was not significant. These findings were again consistent with a data-driven relationship between self-evaluations and growth mindsets (Koriat 2006).

We also tested how creative potential assessed by judges correlated with creative mindsets. For growth mindsets, the correlations were not significant in both studies. This could be interpreted by means of suggesting that when individuals activate knowledge structures of malleable mindsets, they might pay less attention to creative potential or performance (Plaks 2017). For fixed mindsets, study 1 showed a negative relationship with the creative potential of task 1 and a non-significant relationship with the creative potential of task 2. Conversely, in study 2, we observed a nonsignificant relationship with the creative potential of task 1 and a significant negative relationship with the creative potential of task 2. The pattern of results was not as consistent as we would have hoped for fixed mindsets. While the negative relationship between fixed mindsets and creative potential of task 1 was promising, we were not able to replicate this relationship in study 2. It is worth mentioning that in previous studies (Karwowski et al. 2019a), it is common to find non-significant relationships between creative mindsets and potential. One possible explanation is that the first creative task of study 1 was more informative than the first creative task of study 2. Future research could conduct both studies using a different sample but the same creative task to shed light on these and other inconsistent findings.

The overall model showed partial support for the idea that creative mindsets act as knowledge structures sensitive to variations in different cognitive and metacognitive indicators. Future research should conduct additional studies to examine the consistency and adequacy of conceptualizing creative mindsets as knowledge structures with different levels of activation.

In sum, our results provide preliminary evidence for metacognitive feelings, creative potential, and self-evaluations as relevant sources of information for creative mindsets when assessed in creative action. The results have implications for conceptualizing mindsets as a form of creative awareness, for models of situated cognition, and for examining mindsets as data-driven knowledge structures.

### 4.2. Limitations and Future Directions

Our main goal was to show that creative mindsets, as a form of knowledge structure, were sensitive to variations in creative potential, self-evaluations, and metacognitive feelings. Even though we found partial support for our propositions, we only presented results from two new studies in which the examination of mindsets was conducted in creative action. Consequently, our efforts need to be replicated. Another limitation is that we did not collect additional consequences, beyond a second creative potential score, of variations in creative mindsets. Future studies could assess effort (Mrazek et al. 2018) as an additional motivational variable likely to vary as a function of creative potential, metacognitive feelings, and creative mindsets after performing a divergent thinking task while knowing that a second task is ahead. Another relevant limitation was that finding a significant correlation between the three proposed sources of information and creative mindsets provides initial, but not definitive support for our propositions. Future studies could complement our results with experimental designs in which success and failure performance feedback is manipulated and then assess its effect on creative mindsets.

### 4.3. Implications for Education

Our results have implications for education. Through the involvement of students in the process of generating ideas to solve business problems, we, as educators, could create a creative academic environment with the purpose of molding students’ creative mindsets and their expectations about mindsets to improve their creative potential and creative outcomes. Although this investigation was carried out with business students, we would like to highlight that higher education institutions could create creative experiences not limited to business students, but also for students from different disciplines (health sciences, engineering, arts and design, among others). The creation of such opportunities can offer students the chance to creatively find ways of applying their academic education to the solution of problems. Creative mindsets are malleable (Karwowski et al. 2019b), trainable (Mrazek et al. 2018), and, as shown in our studies, sensitive to variations in creative performance, self-evaluations, and metacognitive feelings because they are knowledge structures that adjust to context. Explaining to students the situated nature of beliefs and judgments might help them deal with experiences of success and failure in the difficult task of generating, evaluating, and selecting ideas to solve problems without a single, right solution.

Our results also have implications for teachers’ education and training. Providing teachers with the perspective of situated creative mindsets should allow them to respond more effectively to students’ experiences of success and failure during creative endeavors. As recommended by creativity scholars and other scientists, failure could represent a great opportunity to generate novel ideas and solutions to problems (Beghetto 2021; Firestein 2015). Fluctuations of beliefs as a function of failure are expected, and this understanding might allow teachers and students to continue their efforts regardless of expected and unexpected negative results and be able to frame failure as an opportunity.

In sum, our study conceptualized creative mindsets as knowledge structures and an awareness of the nature of creativity. These beliefs were sensitive to variations in creative potential, self-evaluations, and metacognitive feelings, suggesting that creative mindsets might play a relevant role in creative metacognition and creative action (Lebuda and Benedek 2023). Future research could continue exploring metacognitive antecedents of creative mindsets under a situated cognition perspective of creative problem-solving.

## Figures and Tables

**Table 1:** Sequence of procedure for studies 1 and 2.

Sequences	Divergent Thinking Task	Metacognitive Feelings	Self-Evaluations	Creative Mindsets	Second Divergent Thinking Task
Study 1	First	Second	N/A	Third	Fourth
Study 2	First	Second	Third	Fourth	Fifth

NA = Not applicable. Variables were not measured.

**Table 2:** Summary of results.

Study 1	Coefficient	*p*-Value
Metacognitive feelings on creative potential task 1	0.13	0.28
Growth mindset on creative potential task 1	0.2	0.21
Fixed mindset on creative potential task 1	−0.45	<.001
Growth mindset on metacognitive feelings	0.18	0.01
Fixed mindset on metacognitive feelings	−0.006	0.94
Creative potential task 2 on growth mindset	0.12	0.62
Creative potential task 2 on fixed mindset	0.32	0.41
Creative potential task 2 on metacognitive feelings	−0.13	0.46
**Study 2**		
Self-evaluations on creative potential task 1	0.07	0.23
Metacognitive feelings on creative potential task 1	0.10	0.17
Metacognitive feelings on self-evaluation	0.74	<.001
Growth mindset on creative potential task 1	0.07	0.23
Growth mindset on metacognitive feelings	0.19	0.03
Growth mindset on self-evaluations	0.42	<.001
Fixed mindset on creative potential task 1	0.02	0.80
Fixed mindset on metacognitive feelings	0.42	<.001
Fixed mindset on self-evaluations	−0.12	0.30
Creative potential task 2 on growth mindset	0.13	0.22
Creative potential task 2 on fixed mindset	−0.36	<.001
Creative potential task 2 on metacognitive feelings	0.04	0.76

## Data Availability

The data is available upon request from first author.
